# P10-05 Evaluation of a MOOC on Heath Promotion in sports club

**DOI:** 10.1093/eurpub/ckac095.144

**Published:** 2022-08-29

**Authors:** Benjamin Tezier, Stacey Johnson, Anne Vuillemin, Florence Rostan, Fabienne Lemonnier, Francis Guillemin, Aurélie Van Hoye

**Affiliations:** 1 APEMAC, Université de Lorraine, Nancy, France; 2 Institute de Cancérologie de l'Ouest, Centre René Gauducheau, Nantes, France; 3 Université Côte d'Azur, LAMHESS, France; 4 Santé publique France, Paris, France; 5 Physical Education and Sport Sciences Department, University of Limerick, Limerick, Ireland

**Keywords:** Health promotion, sports clubs, education

## Abstract

**Background:**

The acquisition of health promotion (HP) skills and knowledge is essential for interventions development [1,2]. Considering the increasing recognition of the potential of HP in sports clubs (SC) [3] and the number of HP interventions [4,5], the development of training to optimize their implementation is important. To this end, the MOOC (Massive Open Online Course) PROSCeSS (PROmotion de la Santé dans les Clubs de SportS) was developed. The objective of this work was to evaluate the learning process of the MOOC, its acceptability and its effectiveness on the HP knowledge and on the abilities to implement the learning.

**Methods:**

Questionnaires were sent to participants before and after the training between November and February 2022. The RE-AIM model, measuring reach (affected audience) of the training, effectiveness (knowledge gained), adoption (motivations to participate in the course), implementation (use of learning), and maintenance (long-term use of learning) was used to structure the study. Descriptive and multivariate statistics were performed using SPSS 23.0 software.

**Preliminary Results:**

Of the 2000 learners, 21% completed the pre-MOOC questionnaire and 5% completed the post-MOOC questionnaire. Among non-exclusive categories, 32% of learners were SC coaches, 26% were managers, 43% were practitioners (on average for 10 years) and 46% were HP or sport professionals. A paired sample t-test showed an 11% increase in the post-MOOC knowledge questionnaire score compared to the pre-MOOC questionnaire, as well as a 10% increase in confidence in implementing HP actions. 48% of learners were “completely satisfied” with the MOOC and 47% “quite satisfied”. The strategies of participatory approach, communication and objective were considered as the most important and feasible while the lack of time, financial and human resources were considered the main barriers to learning's implementation.

**Conclusions:**

SC actors seem to be interested in HP and want to be trained. This MOOC appears to be an effective solution for acquiring knowledge in HP. The results provide indications for the development of strategic tools and inform on strategies recognized as effective and feasible to implement HP projects and face the problems encountered by the SC.

